# Distress financing on inpatient health expenditure across States in India

**DOI:** 10.1186/s12913-025-13842-y

**Published:** 2025-12-13

**Authors:** Kamlesh Meena, Sanatan Nayak

**Affiliations:** Department of Economics, Babasaheb Bhimrao Ambedkar University, Lucknow, India

**Keywords:** Out-of-pocket expenditure, Distress financing, Coping mechanism, Inpatient healthcare, Rural and urban India

## Abstract

**Background:**

Out-of-pocket (OOP) healthcare expenditures remain a significant burden and are a leading cause of distress financing, particularly among socioeconomically disadvantaged households in India. Despite recent reductions in OOP spending, many families continue to experience financial hardship when seeking inpatient care. The study aims to examine state-level disparities and key socioeconomic determinants influencing reliance on distress financing as a coping strategy.

**Methods:**

Data from the 75th round (2017–18) National Sample Survey Organization (NSSO), covering 113,823 Indian households, were analyzed. Distress financing was defined as any non-income/savings method used to pay for inpatient care. Logistic regression was conducted to identify key socio-economic and health-related determinants at national and state levels.

**Results:**

Scheduled Castes (SC) and Scheduled Tribes (ST), as well as households with lower income and education levels, exhibited significantly higher reliance on distress financing. States such as Uttar Pradesh, West Bengal, Maharashtra, and Rajasthan demonstrated the greatest incidence of distress financing, particularly in rural areas. Even among insured households, distress financing persisted, especially in rural settings. Regression analysis identified social group, income quintile, education, occupation, household size, and health insurance coverage as significant predictors of distress financing.

**Conclusions:**

Distress financing for inpatient healthcare persists as a major challenge in India, despite a downward trend in OOP expenses. The burden is disproportionately higher among vulnerable groups and in states with weaker public healthcare systems. Policymakers should prioritize targeted insurance coverage, health infrastructure strengthening, and need-based financial protection for high-risk groups to reduce the incidence of distress financing and increase equity in healthcare access

## Introduction

Healthcare systems across the globe aim to offer high-quality accessibility and affordability for all [1]. However, despite continuous progress, out-of-pocket (OOP) healthcare expenses remain a major burden, significantly contributing to distress financing, particularly in low- and middle-income countries [2]. This burden is evident as OOP expenditures often exceed 40% of total health spending in these regions, pushing many households into poverty and forcing them to resort to distress financing methods such as borrowing or selling assets to cover medical costs. The high reliance on OOP payments not only limits access to necessary care but also exacerbates financial vulnerability, such as distress financing.^1^

Distress financing affects mainly poor people and can create a long-term financial burden. It is common in developing countries such as Argentina, Tanzania, India, and China [3–5]. In India, as of 2021–2022, OOP expenditures represented approximately 39.4% of total health spending, reflecting a decreasing trend from 64.2% in 2013–2014 (National Health Account 2024). However, this figure remains considerably higher than the global average of 18.1% as of 2019, indicating persistent challenges in healthcare financing within the country [6].

Numerous studies indicate that high OOP expenses significantly increase poverty levels and indebtedness, particularly affecting rural and low-income households [7, 8, Kane et al. 2023]. For example, [9], reported that approximately 60% of rural households and 50% of urban households with inpatient cases resort to distress financing methods, such as borrowing from friends and relatives, taking loans, or selling assets. This reliance on distress financing adversely affects household living standards, leading to altered consumption patterns, income loss due to loan repayments, and diminished productive capacity from asset liquidation [10–12].

The economic burden associated with high OOP expenditures is particularly pronounced in the context of healthcare in India. High OOP costs often compel families to resort to distress financing methods to cover medical expenses, thereby exacerbating financial strain. Estimates suggest that healthcare costs push approximately 25 million households into poverty annually [13–16]. The increasing incidence of OOP health expenditures can be attributed to inadequate health insurance coverage and poor-quality public healthcare services [17]. In many developing countries, households frequently resort to informal strategies such as seeking help from family and friends, selling assets, or borrowing from moneylenders to mitigate the financial impact of health crises due to inadequate access to credit and insurance [18]. The issue of distress financing for inpatient health expenditures in India, particularly in states such as Uttar Pradesh, highlights significant disparities in healthcare access and financial protection [19, 20].

Although several studies in India have examined distress financing using earlier NSS rounds (60th, 71st) and focused primarily on national averages [14, 21], comprehensive state-level comparisons using the latest NSS 75th round (2017–18) remain limited. Moreover, existing research often overlooks how socioeconomic and health disparities interact with emerging government initiatives, such as Ayushman Bharat–Pradhan Mantri Jan Arogya Yojana (AB-PMJAY), which began around the same period. This study fills that gap by providing an updated, comparative, and disaggregated analysis of distress financing across states and residence types (rural–urban), identifying vulnerable populations and assessing the persistence of financial hardship despite health reforms. The findings aim to generate evidence for designing targeted financial protection policies at both the state and national levels.

Specifically, this study investigates the impact of socioeconomic and health variables on the likelihood of employing distress financing to cover out-of-pocket health expenditures among inpatients across Indian states. The paper is organized into four sections: following this introduction, Sect. 2 outlines the methodology, data sources, and variables used; Sect. 3 presents the key results; and Sect. 4 discusses the findings, concludes, and offers policy recommendations.

## Methodology

### Source and nature of the data

This article uses unit- level data from the 75th Round (2017–18) of the National Sample Survey Organization (NSSO) on ‘Key Indicators of Social Consumption in India: Health.’ The survey was conducted entirely in the union of India, covering 1,13,823 households across major states and union territories. The objective of the survey was to collect quantitative data on the health sector of India. In this round, a two-stage stratified sampling method was adopted, with census villages as the first-stage units (FSUs) for rural areas and urban blocks for urban areas, while households were captured as the second-stage units. The survey was conducted during the 75th round from during June 2017 to July 2018. It gathered information on various sources of finance employed to cope with out-of-pocket (OOP) health expenditures. These sources of finance are categorized in various ways, such as household income/savings, borrowings, sale of physical assets, contributions from friends and relatives, and other sources. The sources of finance are categorized under “major source of finance”. In this study, households were used as the unit of analysis, and all estimates were adjusted according to their respective weights.

### Variables used

We have used a key outcome variable in binary format in the way ‘whether a household resorts to distress financing’ (borrowings, contributions, sale of assets, and other sources) as a coping mechanism to meet out the expenditures for inpatient healthcare (Table 1). The percentage share of distress financing is derived by using values of distress financing out of total OOPE which can be mathematically derived as:

$$\begin{aligned}&Percentage{\text{ }}Share{\text{ }}of{\text{ }}DF\cr &= \frac{{Distress{\text{ }}Financing}}{{Out{\text{ }}of{\text{ }}Pocket{\text{ }}Expenditure}}*100\end{aligned}$$

**Table 1:** Description of variables used in logistic regression analysis

Variables	Description of variables	Source	Categorization of Variables	Mean	Standard Deviation
Dependent Variable (Distress Financing)	This variable indicates whether a household has engaged in distress financing due to healthcare costs. It is crucial for understanding the financial burden of healthcare on families	[22]	Yes = 1, No = 0	0.14	0.35
Independent variables					
Gender	This variable captures the gender of the household head. Gender dynamics can influence access to resources and decision-making regarding healthcare financing	[23]	Male = 0, Female = 1	1.49	0.5
Religion	The religious affiliation of the household can affect cultural attitudes toward health-seeking behavior and financial practices related to healthcare	[24]	Hinduism = 0, Islam = 1, Others = 2	1.2	0.41
Caste	Caste classification can reveal socioeconomic disparities within households. Marginalized castes often face additional barriers to accessing healthcare and financial resources	[Kumar et al. 25]	General = 0, SC = 1, ST = 2, OBC = 3	1.75	0.43
Marital Status	Marital status may influence household economic stability and support systems during health crises. For instance, unmarried individuals may lack shared financial resources	[26]	Married = 0, Unmarried = 1, Widowed/Divorced = 3	1.53	0.57
Household Size	Larger households may experience greater financial strain due to increased healthcare needs and expenses. This variable helps assess the impact of family structure on distress financing	[27]	less than 5 = 0, more than 5 = 1	1.67	0.46
Occupation	The type of occupation reflects income stability and access to health benefits. Casual or agricultural workers are typically more vulnerable to distress financing due to irregular income	(Giannetti et al. 2014)	Regular/Salaried = 0, Casual/Agriculture = 1, Others = 3	2.55	2.17
Consumption Expenditure Group	This categorization indicates the economic status of households. Those in lower expenditure groups are more likely to incur catastrophic health expenditures leading to distress financing	[28]	Poorest = 0, Second = 1, Middle = 2, Fourth = 3, Richest = 4	2.59	1.38
Education	Education levels influence health literacy and employment opportunities. Higher education correlates with better economic outcomes and reduced reliance on distress financing	[29]	Higher Education = 0, Illiterate = 1, Upper Primary = 2, Higher Secondary = 3,	1.97	0.93
Household Member using private hospital facility	This variable assesses whether households utilize private healthcare services that often come with higher costs. Increased reliance on private facilities can lead to higher rates of distress financing	[30]	No = 0, Yes = 1	1.82	0.37
Covered under medical insurance	Having medical insurance is critical for reducing out-of-pocket expenses. Households without insurance are more likely to resort to distress financing during health emergencies	(Arviana et al. 2024)	No = 0, Yes = 1	1.98	0.13
Household member suffering from chronic ailment	Chronic illnesses can lead to ongoing healthcare costs that strain household finances. This variable is essential for understanding the persistent financial burdens faced by families with sick members	[31]	No = 0, Yes = 1	1.97	0.16

*Source* Various sources as mentioned above

The likelihood of a household utilizing a particular source of financing from distress financing sources or income/savings is influenced by various socioeconomic and health-related factors [Kumar et al. 2015; 14]. Key independent variables, such as religion, sex, social group, marital status, household size, occupation, consumption expenditure, education level, health insurance coverage, and the presence of chronic illnesses within households, were included in this study (Table 1). The population is predominantly Hindu (80%), with Muslims and other religions accounting for 20% of the population (Registrar General of India 2011). Household size is considered to reflect coping mechanisms by dividing it into as small versus larger families, while household type is distinguished between those engaged in agriculture or casual labor and those with regular salaried jobs. Given the challenges in obtaining reliable income data, this study has uses reported household monthly per capita consumption expenditure (MPCE) as a proxy variable to reflect economic status. Moreover, this variable was categorized into wealth quintiles such as poorest, poorer, middle, richer and richest. Furthermore, health-related variables, including the use of private healthcare facilities, chronic ailments, and medical insurance coverage, are also significant in determining the likelihood of using distress financing.

### Inequality measurement

To assess socioeconomic inequalities in the use of different sources of financing, the concentration index (CI) and concentration curve (CC) are used [32]. The CC plots the cumulative percentage of households, ranked by their socioeconomic status, on the x-axis against the cumulative percentage of households utilizing a particular source of financing on the y-axis. If the distribution of a financing source is equal across all socioeconomic groups, the CC aligns with the 45° line of equality. However, if the source of financing is concentrated among wealthier groups, the curve will lie below the equality line; if it is concentrated among poorer groups, it will lie above the equality line. The further the curve deviates from the line of equality, the greater the inequality.

The concentration index (CI) is derived from the area between the concentration curve and the line of equality and ranges from − 1 to + 1. A negative CI value indicates that the source of financing is disproportionately concentrated among poorer households, whereas a positive value suggests a concentration among wealthier households. The CI is calculated via the following equation:

Formula:

$$\begin{aligned}&Ln{\text{ }}\left( {Pi/1 - Pi} \right){\text{ }} =\cr & {\text{ }}\left( {p{\text{ }}1{\text{ }}L{\text{ }}2{\text{ }}-{\text{ }}p{\text{ }}2{\text{ }}L{\text{ }}1} \right){\text{ }} + {\text{ }}\left( {p{\text{ }}2{\text{ }}L{\text{ }}3{\text{ }}-{\text{ }}p{\text{ }}3{\text{ }}L{\text{ }}2} \right){\text{ }} \cr &+ {\text{ }} \ldots .{\text{ }} + {\text{ }}\left( {p{\text{ }}t - 1{\text{ }}L{\text{ }}t{\text{ }}-{\text{ }}p{\text{ }}t{\text{ }}L{\text{ }}t - 1} \right)\end{aligned}$$

Here, pt represents the cumulative percentage of households ranked by consumption expenditure in group t, and Lt is the corresponding ordinate of the concentration curve.

### Factors determining the likelihood of distress financing

Logistic regression is used to understand the relationship between socioeconomic characteristics and the likelihood of using different sources of financing for inpatient treatment. The analysis emphasizes inpatient care because there is significant reliance on distress financing, as health expenditures normally become very high. The logistic regression model can be represented as follows:

$$\begin{aligned}Ln = &\beta 0 + \beta 1X1 + \beta 2X2 + \beta 3X3 + \beta 4X4 \cr & \quad+ \beta 5X5 + \beta 6X6 + \beta 7X7 + \beta 8X8 \cr & \quad+ \beta 9X9 + \beta 10X10 + \beta 11X11 + \mu i\end{aligned}$$

Logistic regression was chosen for its ability to estimate the probability of distress financing based on multiple categorical and continuous predictors simultaneously. This method is widely used in health economics for analyzing binary outcomes such as catastrophic or distress expenditures [14, 33, 34]. To ensure model reliability, Variance Inflation Factor (VIF) was computed to test for multicollinearity among explanatory variables, and Average Marginal Effects (AMEs) were estimated to interpret the magnitude of influence of each factor. Furthermore, the Concentration Index (CI) and Concentration Curve (CC) were used to capture the inequality dimension of distress financing across economic quintiles. This mixed approach—combining regression and inequality analysis—offers both statistical robustness and policy relevance by linking determinants with distributional impacts.

The probability of using distress sources as a coping mechanism is represented by *P*, while 1 − *P* indicates the probability of not using these sources. The independent variables in the model are defined as follows: X_1_ represents gender, X_2_ denotes religion, X_3_ indicates social group, X_4_ refers to marital status, X_5_ corresponds to household size, X_6_ denotes occupation, X_7_ indicates consumption expenditure groups, X_8_ represents education level, X_9_ signifies whether a household member uses a private healthcare facility, X_10_ shows whether the household is covered under medical insurance, and X_11_ indicates whether a household member suffers from a chronic ailment. The term µ_i_ represents the random disturbance component, whereas β_1_ to β_11_ are the coefficients that need to be estimated.

## Analysis of results

### Distress financing across regions around the globe

**Fig. 1:**
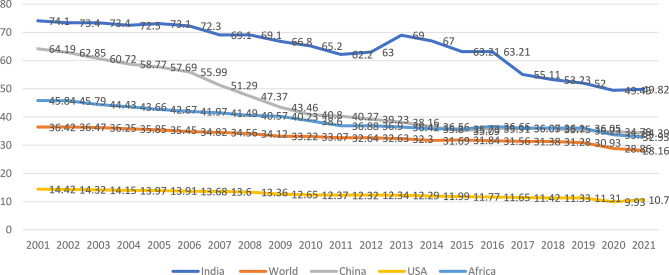
Share of OOPE at the global level. Source WHO, 2023

Figure 1 illustrates the trends in out-of-pocket expenditures (OOPEs) as a percentage of total health spending across different regions—India, the world average, China, the USA, and Africa—over the period from 2001–2021. The data reveal a consistent decline in OOPE globally, with varying rates of reduction among regions. India has experienced a significant decrease in OOPE, starting at approximately 74% in 2001 and declining to approximately 39.7% by 2021. Despite this improvement, India’s OOPE remains higher than the global average, which decreased approximately about 36% in 2001 to approximately 18% in 2021. This indicates India’s laggardness in healthcare financing compared with global standards. China exhibited a sharp reduction in OOPE during the same period, dropping from over 64% in 2001 to approximately 28.6% by 2021. This trend highlights China’s effective healthcare reforms aimed at reducing financial burdens on households. In contrast, the USA maintained relatively low OOPE levels throughout the period, starting at approximately 14% in 2001 and stabilizing near 10% by 2021. This reflects the country’s robust insurance coverage and healthcare financing mechanisms. Compared with those in other regions, Africa’s OOPE levels remain high, although there was a gradual decline from approximately 45% in 2001 to around 40.82% in 2021to approximately. This reveals persistent ongoing challenges in healthcare accessibility and affordability across the continent. Overall, while global OOPE trends indicate progress toward reducing financial barriers to healthcare access, despite significant disparities persist across regions, with India and Africa facing greater financial burdens than developed nations do.

### Distress financing across states in India: extent and dimensions

Table 2 presents an analysis of distress financing for inpatient care across Indian states, which is based on the 75th round of the National Sample Survey (2017–18). It reveals considerable disparities by area of residence (rural/urban), social group (ST, SC, OBC, general), and household wealth status (consumption expenditure). The national average of distress financing stands at 2.78%, but statewise analysis reveals significant variations. In rural areas, the highest incidence of distress financing is observed in Uttar Pradesh (19.1%), followed by West Bengal (8.46%), Maharashtra (7.96%), and Rajasthan (7.54%), indicating greater financial strain in these regions. These states generally report high out-of-pocket expenditures and limited health insurance coverage in rural populations. In contrast, rural populations in Sikkim (0.04%), Nagaland (0.07%), and Goa (0.06%) reported the lowest levels of distress financing, indicating relatively better financial protection or access to subsidized public healthcare. In urban areas, the burden of distress financing is particularly high in Maharashtra (12.91%), Tamil Nadu (7.7%), and Delhi (5.7%), likely due to greater dependency on private healthcare services with higher costs. However, states such as Sikkim (0.02%), Arunachal Pradesh (0.05%), and Dadra & Nagar Haveli (0.04%) report very low urban distress financing, suggesting better cost mitigation or lower healthcare utilization.

**Table 2:** Utilization of various sources of financing, social group- wise, and income group- wise distress financing in inpatient care among rural and urban areas in Indian states, 2017

State	Rural		Urban		Rural (Distress Financing)				Urban (Distress Financing)				Rural (Distress Financing)					Urban (Distress Financing)				
	Income and Saving	Distress Financing	Income and Saving	Distress Financing	ST	SC	OBC	General	ST	SC	OBC	General	Poorest	Poorer	Middle	Richer	Richest	Poorest	Poorer	Middle	Richer	Richest
Jammu & Kashmir	99	1	99.36	0.64	1.27	0.47	0.39	2.61	0.68	0.87	0.08	1.17	0.57	1.01	1.13	1.54	1.4	0.56	0.67	0.5	0.66	0.7
Himachal Pradesh	99.2	0.8	99.88	0.12	0.48	1.14	0.23	1.77	0.12	0.19	0.03	0.21	0.45	0.78	0.94	1.18	1.25	0.11	0.1	0.07	0.13	0.17
Punjab	98.03	1.97	97.38	2.62	0.14	3.65	0.84	3.36	0.09	4.53	0.93	3.96	0.44	0.91	1.86	3.69	8.06	1.31	1.23	2.01	2.18	4.08
Chandigarh	99.87	0.13	99.74	0.26	0.02	0.17	0.12	0.16	0	0.41	0.07	0.43	0.06	0.07	0.05	0.36	0.36	0.22	0.13	0.17	0.14	0.46
Uttarakhand	99.33	0.67	99.47	0.53	0.2	0.6	0.42	1.41	0.15	0.43	0.38	0.77	0.41	0.55	0.94	0.72	1.24	0.31	0.37	0.71	0.54	0.54
Haryana	97.26	2.74	97.25	2.75	0.15	4.1	1.94	4.11	0.7	4.93	1.33	3.66	0.65	1.81	3.81	4.54	7.63	1.16	1.96	1.96	2.34	4.18
Delhi	99.67	0.33	94.3	5.7	0.06	0.33	0.41	0.28	8.17	7.34	2.96	7.97	0.13	0.21	0.4	0.49	0.96	1.64	2.28	6.04	4.48	8.6
Rajasthan	92.46	7.54	95.08	4.92	10.7	8.44	8.44	3.59	4.87	4.39	5.99	3.93	3.82	6.3	8.9	11.95	14.17	3.44	4.08	2.67	5.12	6.69
Uttar Pradesh	80.9	19.1	87.42	12.58	1.48	23.67	21.67	16.9	3.07	11.41	14.77	11.24	20.06	19.82	18.14	18.33	17.19	18.9	14.77	12.27	12.74	10.21
Bihar	91.44	8.56	97.79	2.21	1.55	8.52	12.06	4.54	0.9	1.81	3.24	1.3	14.02	9.96	5.93	2.35	1.02	5.58	3.68	2.58	1.77	0.93
Sikkim	99.96	0.04	99.98	0.02	0.18	0.02	0.05	0	0.1	0.03	0.03	0.01	0.03	0.04	0.06	0.06	0.02	0.04	0.01	0.04	0.03	0.01
Arunachal Pradesh	99.88	0.12	99.95	0.05	1.08	0	0	0.08	1.14	0.01	0	0.05	0.16	0.09	0.1	0.12	0.1	0.15	0.08	0.05	0.04	0.02
Nagaland	99.93	0.07	99.93	0.07	0.7	0.02	0	0.01	2.34	0.01	0.01	0.01	0.02	0.1	0.11	0.09	0.05	0.07	0.05	0.07	0.07	0.09
Manipur	99.77	0.23	99.76	0.24	1.27	0.01	0.22	0.02	1.32	0.16	0.37	0.04	0.21	0.25	0.24	0.24	0.17	0.36	0.43	0.29	0.31	0.07
Mizoram	99.94	0.06	99.87	0.13	0.66	0	0	0	4.61	0.01	0	0	0.03	0.05	0.08	0.11	0.12	0.05	0.02	0.07	0.09	0.24
Tripura	99.51	0.49	99.78	0.22	2.08	0.44	0.16	0.55	0.45	0.56	0.07	0.24	0.53	0.51	0.49	0.45	0.39	0.23	0.27	0.36	0.21	0.13
Meghalaya	99.71	0.29	99.89	0.11	2.59	0.02	0.01	0.14	2.74	0.01	0.01	0.08	0.08	0.32	0.48	0.46	0.18	0.02	0.05	0.08	0.14	0.16
Assam	97.92	2.08	99.35	0.65	3.1	0.65	1.45	4.3	1.7	0.77	0.37	0.85	2.57	2.56	1.8	1.4	0.83	0.74	0.82	0.59	0.65	0.61
West Bengal	91.54	8.46	91.59	8.41	5.79	8.97	3.53	18.9	2.87	10.32	2.35	14.86	9.67	9.81	8.29	6.51	4.06	10.94	10.99	8.95	8.38	6.6
Jharkhand	97.91	2.09	98.47	1.53	4.72	1.72	2.41	0.72	3.75	1.34	1.82	1.12	3.05	2.38	1.18	1.24	1.31	2.89	1.88	1.74	1.38	1.04
Odisha	95.96	4.04	98.34	1.66	9.24	4.54	3.31	2.87	4.88	2.62	1.26	1.5	8.81	2.32	1.74	0.97	0.99	4.32	2.9	1.44	1.15	1.02
Chhattisgarh	97.85	2.15	98.72	1.28	8.08	1.31	2.19	0.42	6.13	1.38	1.27	0.92	4.32	1.72	1.02	0.57	0.34	2.91	2.21	1.09	0.65	1.1
Madhya Pradesh	93.93	6.07	94.6	5.4	14.57	5.79	5.96	3.04	8.94	5.43	6.48	3.93	7.77	6.2	5.87	3.73	3.75	7.65	7.75	6.5	4.64	3.98
Gujarat	95.67	4.33	94	6	10.69	2.47	4.34	3.43	9.52	3.06	5.04	7.98	1.35	2.96	6.2	8.7	7.11	2.29	2.14	4.63	6.1	8.95
Daman &Diu	100	0	99.98	0.02	0	0	0	0	0.03	0	0.01	0.03	0	0	0	0.01	0.01	0.01	0.01	0.02	0.01	0.03
Dadra & Nagar Haveli	99.98	0.02	99.96	0.04	0.24	0	0	0	0.38	0.01	0.02	0.06	0.01	0.05	0.04	0.02	0	0.01	0.07	0.08	0.05	0.03
Maharashtra	92.04	7.96	87.09	12.91	8.38	5.09	6.94	12.53	11.71	12.94	9.24	17.11	7.51	7.66	8.88	8.12	7.92	10.71	9.89	12.5	14.72	13.41
Andhra Pradesh	96.15	3.85	95.82	4.18	2.97	3.9	3.91	4.06	3.31	3.84	5.25	3.18	3.59	4.44	4.31	3.79	2.3	4.6	6.09	4.83	5.35	2.25
Karnataka	95.4	4.6	93.98	6.02	3.49	4.99	4.93	4.05	7.97	5.65	6.86	5.08	3.18	6.59	4.81	5.11	3.47	4.02	6.38	6.19	5.83	6.47
Goa	99.94	0.06	99.73	0.27	0.04	0.01	0.02	0.2	0.11	0.06	0.14	0.5	0	0.04	0.09	0.13	0.17	0.13	0.25	0.35	0.36	0.19
Lakshadweep	100	0	99.99	0.01	0.02	0	0	4.69	0.43	0	0	0	0	0	0	0.01	0	0	0	0.01	0.01	0.02
Kerala	95.89	4.11	92.85	7.15	0.66	1.69	5.56	4.96	0.57	2.86	11.57	4.33	1.47	2.78	5.25	7.46	9.19	6.17	5.38	9	9.5	5.3
Tamil Nadu	96.04	3.96	92.3	7.7	0.71	5.56	5.73	0.27	3.92	9.11	13.74	0.63	3.32	4.85	4.53	3.98	2.64	6.39	9.46	8.7	7.23	7.25
Puducherry	99.82	0.18	99.81	0.19	0.12	0.27	0.22	0.05	0.06	0.25	0.32	0.03	0.13	0.16	0.27	0.23	0.07	0.17	0.21	0.23	0.2	0.17
A & N Islands	99.97	0.03	99.96	0.04	0.09	0	0.02	0.07	0.05	0	0.01	0.08	0.01	0.01	0.03	0.08	0.11	0.01	0	0.03	0.06	0.04
Telangana	98.15	1.85	96.65	3.35	2.5	1.43	2.52	0.63	2.23	3.29	3.98	2.75	1.55	2.69	2.01	1.27	1.41	1.87	3.41	3.18	2.73	4.28
India	97.22278	2.777222	97.22278	2.777222	2.778333	2.7775	2.777778	2.908889	2.778056	2.778611	2.777778	2.778056	2.777222	2.777778	2.777222	2.778056	2.7775	2.777222	2.778333	2.777778	2.7775	2.778333

*Source* Estimated from the unit-level data of the 75^th^ round of NSS data

Distress financing varies notably across social groups. Scheduled Castes (SCs) and Scheduled Tribes (STs) continue to face the highest levels of financial hardship. For example, rural SCs in Uttar Pradesh (23.67%) and rural STs in Madhya Pradesh (14.57%) experienced the greatest burden. These groups, which are historically marginalized, often lack both access to quality healthcare and financial safety nets. Other backward classes (OBCs) represent a large and diverse group, and their distress financing levels lie between the extremes observed for the SC/ST and general categories. Notably, high rates are observed among rural OBCs in Uttar Pradesh (17.54%), Maharashtra (8.23%), and West Bengal (8.14%). These findings highlight that despite being somewhat better off than SC/ST groups on average, OBCs still face substantial financial barriers, particularly in rural settings. Among the general category households, lower overall distress financing is observed, especially in wealthier or better-governed states. However, exceptions exist. For example, urban general households in Maharashtra (17.11%) report notable financial pressure, reflecting how even upper social groups are not fully shielded from healthcare-related financial risk in high-cost states. Moreover, a strong inverse correlation is evident between household wealth and the likelihood of distress financing. Across all regions, the poorest wealth quintiles face the highest burden. For example, among the rural poor, Uttar Pradesh (20.06%), West Bengal (9.67%), and Kerala (9.19%) stand out. Similarly, the urban poor in Tamil Nadu (6.39%) reported substantial financial hardship. The trend is reversed for wealthier households, where distress financing is minimal—often under 1%—indicating their greater ability to pay for healthcare through savings or insurance mechanisms.

The results further reveal that Northern and Central Indian states, especially Uttar Pradesh, Madhya Pradesh, Rajasthan, and Bihar, exhibit the highest distress financing levels, particularly among SCs, STs, OBCs, and the poorest quintile. These states combine weak public healthcare infrastructure, lower insurance coverage, and high poverty levels—making out-of-pocket payments unsustainable for many. In contrast, North Eastern states, Goa, Chandigarh, and Himachal Pradesh show the best financial protection, with distress financing rates below 1% across nearly all categories. This suggests better access to subsidized care or more effective health financing models.

**Fig. 2:**
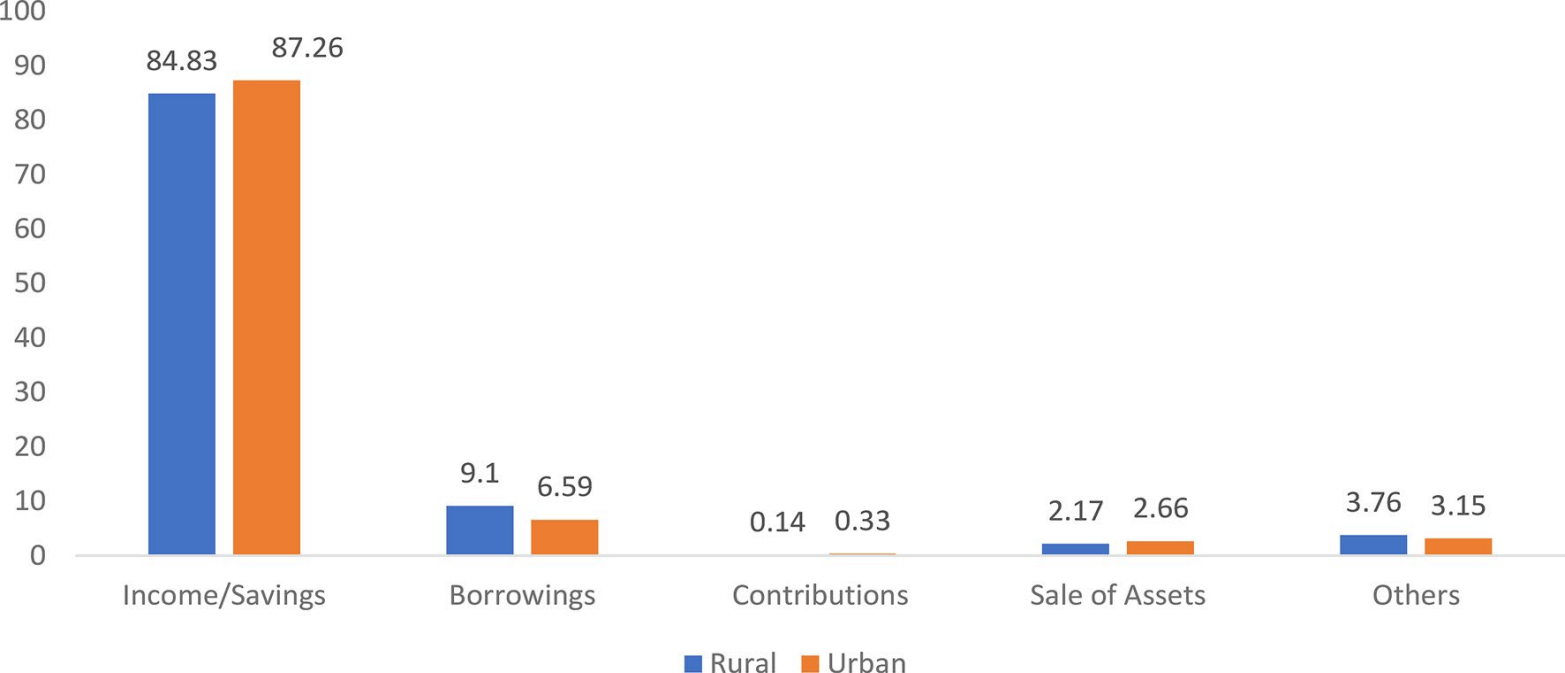
Utilization of various sources of finance as a coping mechanism in the case of inpatient care for rural and urban areas in India, 2017 (in percentages). Source Estimated from the unit-level data of the 75th round of NSS data

The results presented in Fig. 2 illustrate the utilization of various sources of finance as coping mechanisms for inpatient care in rural and urban areas of India from year 2017–2018. In both areas, income/savings were the dominant source, accounting for 84.83% of the total income/saving in rural regions and 87.26% in urban regions. Borrowing was the second most common source but was more prevalent in rural areas (9.1%) than in urban areas (6.59%), indicating greater financial strain in rural households. Sales of assets were slightly more common in urban areas (2.66%) than in rural areas (2.17%), whereas contributions from friends or relatives were minimal in both, although they were slightly greater in urban settings (0.33%) than in rural areas (0.14%). Other sources contributed modestly in both rural (3.76%) and urban (3.15%) areas. The data highlight a greater reliance on out-of-pocket expenditures and informal sources, especially among rural populations.

### Distress financing across the social and economic status of households in India

**Fig. 3:**
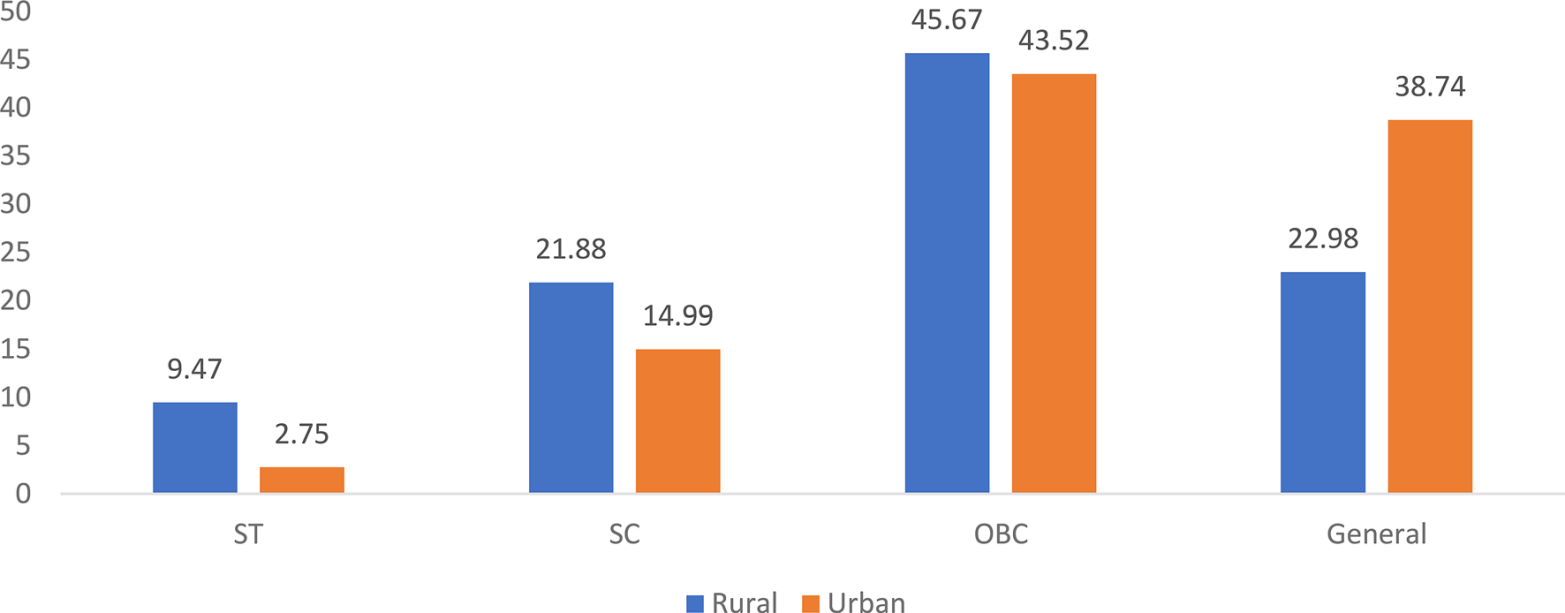
Social group- level distress financing in inpatient care among rural and urban areas, in India, 2017 (in percentages). Source Estimated from the unit-level data of the 75th round of NSS data

Figure 3 displays the social group-wise distribution of distress financing for inpatient care in rural and urban India in 2017. Among rural households, Other backward classes (OBCs) reported the highest incidence of distress financing at 45.67%, followed by the general category at 22.98%, scheduled Castes (SCs) at 21.88%, and scheduled tribes (STs) at 9.47%. In urban areas, OBCs again had the highest share at 43.52%, but the general category closely followed at 38.74%, while SCs and STs reported 14.99% and 2.75%, respectively.

**Fig. 4:**
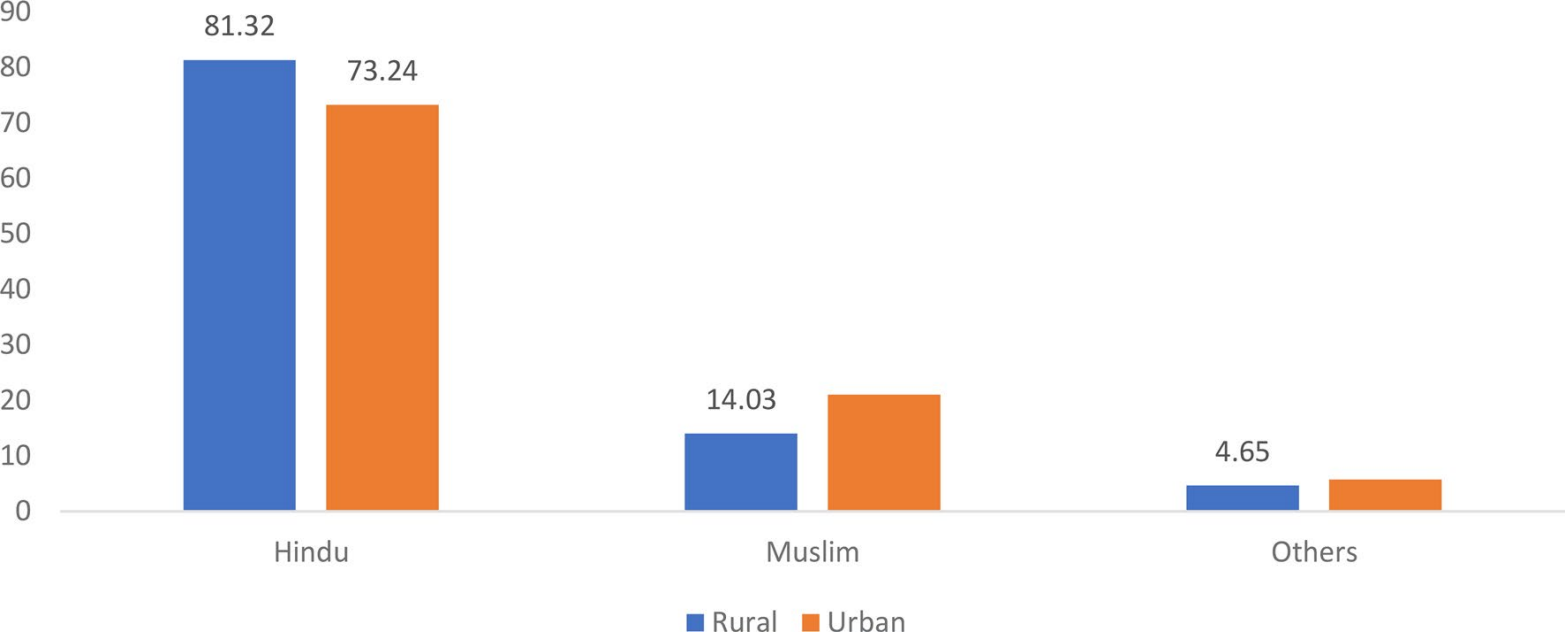
Religion distress financing in inpatient care among rural and urban areas in India, 2017 (in percentages). Source Estimated from the unit-level data of the 75th round of NSS data

Figure 4 shows the religion -wise distribution of distress financing for inpatient care in rural and urban India in 2017. Among rural households, Hindus accounted for the highest share at 81.32%, followed by Muslims at 14.03% and others at 4.65% of total distress financing. In urban areas, the proportion of Hindu households using distress financing decreased to 73.24%, while the share of Muslim households increased to 21.06%, and the share of other households increased to 5.71%. These data suggest that while Hindus constitute the majority of those affected by distress financing, the proportion of Muslims and other religious groups is greater in urban settings, indicating possible disparities in financial resilience or access to healthcare support mechanisms across religious communities.

**Fig. 5:**
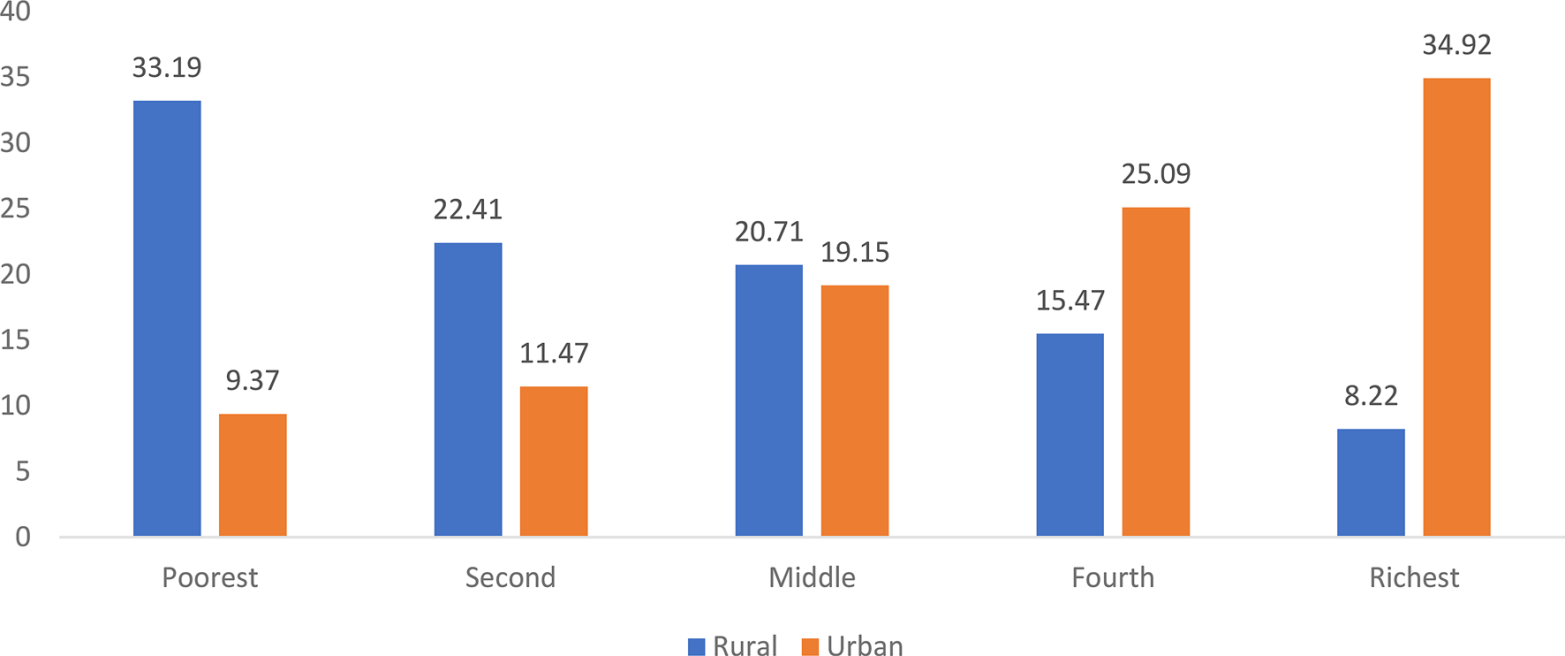
Consumption expenditure-wise distress financing in inpatient care among rural and urban areas in India, 2017 (in percentages). Source Estimated from the unit-level data of the 75th round of NSS data

Figure 5 presents the economic quintile-wise distribution of distress financing for inpatient care in rural and urban India. In rural areas, the burden is highest among the poorest households (33.19%), followed by the second (22.41%), middle (20.71%), and fourth quintiles (15.47%), with the richest facing the least at 8.22% distress financing. This shows a clear decline in distress financing with rising income levels in rural India. In contrast, urban areas exhibit the opposite trend. The richest households reported the highest share of distress financing at 34.92%, followed by the fourth quintile (25.09%) and middle quintile (19.15%). The poorest urban households have the lowest share at 9.37%. This unusual pattern in urban areas may reflect differences in healthcare-seeking behavior, access to credit, or costlier private healthcare utilization by wealthier groups.

**Fig. 6:**
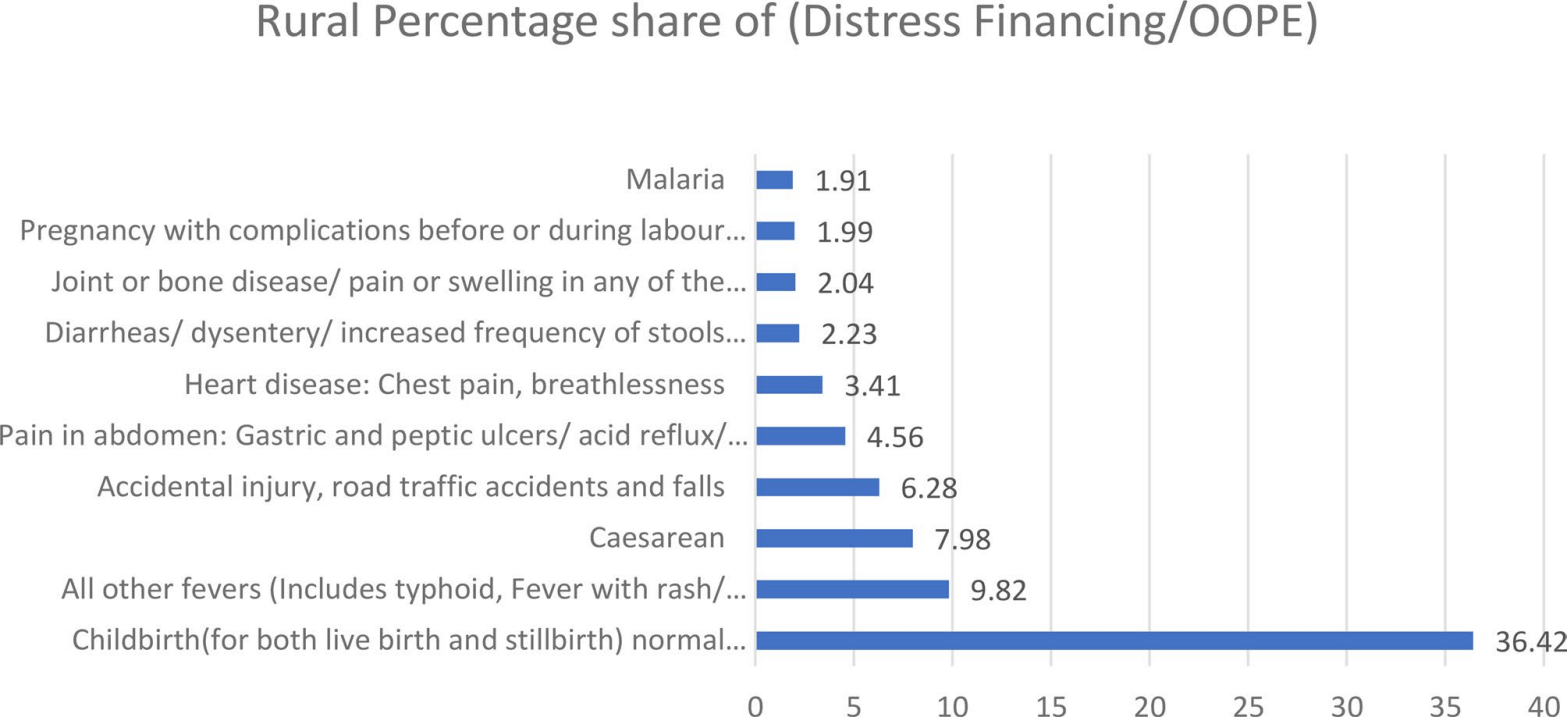
Disease related distress financing for rural areas in India (in percentages). Source Estimated from the unit-level data of the 75th round of NSS data

Figure 6 Analysis of disease related distress financing in rural India. Normal childbirth accounts for the highest share (36.42%), followed by fevers (9.82%), caesarean delivery (7.98%), accidental injury (6.28%), abdominal pain (4.56%), and heart disease (3.41%). Lower but still significant proportions are observed for diarrheal diseases (2.23%), joint or bone ailments (2.04%), pregnancy with complications (1.99%), and malaria (1.91%). The data highlight that maternal health and common infections are leading causes of distress financing, indicating substantial gaps in financial protection and healthcare accessibility.

**Fig. 7:**
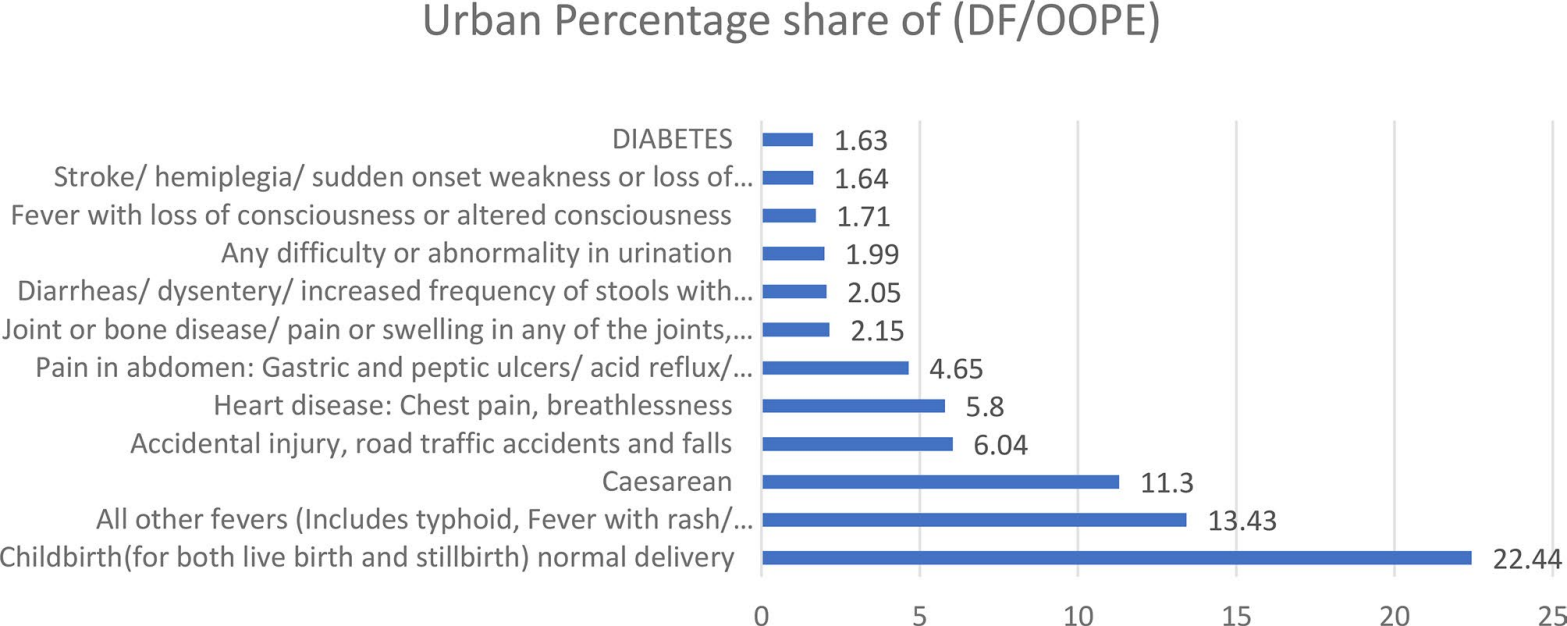
Disease related distress financing for urban areas in India (in percentages). Source Estimated from the unit-level data of the 75th round of NSS data

Figure 7 analysis for urban India indicates that normal childbirth accounts for the highest share of distress financing at 22.44%, followed by all other fevers (13.43%) and caesarean deliveries (11.3%). Other significant contributors include accidental injury (6.04%), heart disease (5.8%), and abdominal pain (4.65%). Lower levels of distress financing are observed for joint or bone diseases (2.15%), diarrheal diseases (2.05%), urinary disorders (1.99%), fever with altered consciousness (1.71%), stroke (1.64%), and diabetes (1.63%). These findings suggest that, similar to rural areas, maternal health and common illnesses are leading contributors to distress financing in urban settings, reflecting ongoing gaps in financial risk protection despite better healthcare infrastructure.

### Factors affecting distress financing across states in India

#### Results of logistic regression (LR)

Table 3 presents the results of a multivariate logistic regression analysis examining the impact of various socioeconomic and health-related factors on the likelihood of using distress financing to cope with out-of-pocket (OOP) health expenditures in rural and urban India. In the term of gender, female-headed households are 1% higher likelihood of using distress financing than male-headed households, with odds ratios of 1.01 in both rural and urban areas. In terms of religion, households practicing Islam are 1% more likely to face distress financing in rural areas but 11% less likely to face it in urban areas than Hindu households are. Those following other religions are 14% less likely to rely on distress financing in rural areas but 7% more likely in urban areas. Caste plays a significant role in the analysis of distress financing, with Scheduled Tribe (ST) households being 20% and 6% less likely in rural and urban areas, respectively, to use distress financing general category households. In contrast, scheduled caste (SC) households are 22% and 21% more likely in rural and urban areas, respectively to rely on distress financing. Similarly, other backward class (OBC) households are 6% and 41% more likely in rural and urban areas, respectively, to depend on distress financing. Marital status also affects distress financing, with unmarried households being 9% and 3% more likely to use it in rural areas and urban areas, respectively, than married households. Widowed or divorced households show even higher odds, being more likely, with 27% and 13% more likely in rural areas and urban areas, respectively, to rely on distress financing. Household size influences this financial burden, as larger households (with more than five members) are 17% and 14% less likely in rural areas and urban areas, respectively, to use distress financing than smaller households are.

**Table 3:** Factors affecting distress financing in India

India				
	Rural		Urban	
	Odd ratio	Z	Odd ratio	Z
Gender				
Male (ref.)	1		1	
Female	1.01	390.00***	1.01	182.28***
Religion				
Hinduism (ref.)	1		1	
Islam	1.01	278.08***	0.89	−1482.89***
Others	0.86	−1796.12***	1.07	656.39***
Caste				
General (ref.)	1		1	
ST	0.80	−3135.19***	0.94	−290.60***
SC	1.22	3995.82***	1.21	2233.59***
OBC	1.06	1467.58***	1.41	5530.17***
Marital Status				
Married (ref.)	1		1	
Unmarried	1.09	2688.74***	1.03	526.60***
Widowed/Divorced	1.27	3288.21***	1.13	1096.75***
Household Size				
<5 members (ref)	1		1	
>5 members	0.83	− 4957.57***	0.86	−2359.77***
Occupation				
Regular/Salaried (ref.)	1		1	
Casual/Agriculture	1.17	2840.90***	1.39	4244.08***
Others	1.13	−1928.16***	1.09	1398.21***
Consumption Exp. Groups				
Poorest (ref.)	1		1	
Second	0.96	− 867.82***	0.96	−362.02***
Middle	0.90	− 2143.77***	0.88	−1256.45**
Fourth	0.74	− 5274.44***	0.74	−3034.95***
Richest	0.71	− 4430.69***	0.55	−5855.73***
Education				
Higher Education (ref.)	1		1	
Illiterate	1.14	1589.34***	1.29	2554.29***
Upper Primary	1.11	1240.80***	1.30	2800.38***
Higher Secondary	1.03	429.05***	1.20	1950.09***
Household Member using private hospital facility				
No (ref.)	1		1	
Yes	1.04	977.83***	1.01	249.51***
Covered under medical insurance				
No (ref.)	1		1	
Yes	1.88	1.5e + 04***	1.61	7481.30***
Household member suffering from chronic ailment				
No (ref.)	1		1	
Yes	1.56	6012.07***	1.43	3687.26***
	Pseudo R2 = 0.0191		Pseudo R2 = 0.0287	

*Source* Estimated from the unit-level data of the 75th round of NSS data

Compared with salaried households, occupational status further impacts distress financing, as households engaged in casual or agricultural work are 17% more likely in rural areas and 39% more likely in urban areas to rely on distress financing. Households engaged in other types of work also show greater dependence on distress financing, with a 13% increase in rural areas and a 9% increase in urban areas. Household income levels, measured through consumption expenditure groups, significantly affect distress financing. Households in higher expenditure categories are progressively less likely to face distress financing, with the richest households being 29% and 45% less likely in rural areas and urban areas, respectively, to rely on it than the poorest households are.

Education plays a crucial role, as illiterate households are 14% and 29% more likely in rural areas and urban areas, respectively, to use distress financing than households with higher education. Those with upper primary education are also more dependent on distress financing, being 11% more likely in rural areas and 30% more likely in urban areas. Similarly, households with higher secondary education are 3% more likely in rural areas and 20% more likely in urban areas to use distress financing. Healthcare access influences the likelihood of distress financing. Households using private hospital facilities are 4% and 1% more likely to be living in rural urban areas, respectively, than are those relying on public healthcare. Moreover, having medical insurance significantly increases the probability of using distress financing, with insured households being 88% more likely in rural areas and 61% more likely in urban areas to resort to distress financing. finally, the presence of chronic illness within a household substantially increases the likelihood of distress financing. Households with a chronically ill member are 56% more likely in rural areas and 43% more likely in urban areas to rely on distress financing than those without chronic illness.

#### Results of the variance inflation factor (VIF)

The variance inflation factor (VIF) is used to check for multicollinearity among the independent variables used in the logistic regression models for distress financing in both rural and urban India. The results in Table 4 reveal that the mean VIF value is 1.97 in rural areas, whereas, it is 1.63 in urban areas. These values are well below the commonly accepted threshold of 10, indicating that multicollinearity is not a significant concern in either model (Gujarati and Sangeetha, 2009). In rural India, the highest VIF is observed for upper primary education (VIF = 6.06) and illiterate (VIF = 5.96), suggesting a moderate correlation between educational categories. However, these values are still within an acceptable range. In urban India, the highest VIF is recorded for the wealthiest households (VIF = 3.62), followed by the fourth and middle expenditure groups, again indicating only moderate multicollinearity related to economic status. Overall, the variance inflation factor (VIF) analysis confirms that the regression results are reliable and that multicollinearity does not significantly distort the estimates in either model.

**Table 4:** Variance inflation factors (VIFs) for independent variables in logistic regression models for distress financing across rural and urban India

Urban			Rural		
Variance	VIF	1/VIF	Variable	VIF	1/VIF
Richest	3.62	0.276	Upper Primary	6.06	0.165082
Fourth	2.955	0.338	Illiterate	5.96	0.167764
Middle	2.524	0.396	Higher Secondary	4.15	0.240939
Upper Primary	2.466	0.406	Casual/Agriculture	2.4	0.416725
Illiterate	2.308	0.433	Other occupation	2.35	0.424828
Higher Secondary	2.065	0.484	OBC	1.69	0.592726
Second	1.981	0.505	SC	1.67	0.597177
Casual/Agriculture	1.328	0.753	Fourth	1.5	0.66883
SC	1.325	0.755	Middle	1.45	0.689954
OBC	1.269	0.788	ST	1.39	0.720537
Other occupation	1.258	0.795	Richest	1.38	0.724313
Unmarried	1.222	0.819	Second	1.37	0.729443
>5 members Household Size	1.216	0.823	>5 members Household Size	1.3	0.768293
Islam religion	1.155	0.865	private_ho ~ 1	1.16	0.864483
private hospital1	1.12	0.893	Unmarried	1.16	0.864708
Widowed/Divorced	1.119	0.894	Islam religion	1.11	0.897974
Yes suffering from chronic ailment	1.102	0.907	Widowed/Divorced	1.1	0.908645
ST	1.069	0.936	Female	1.08	0.929411
Yes covered under medical insurance	1.066	0.938	Yes suffering from chronic ailment	1.08	0.929893
Female	1.054	0.948	Other religion	1.06	0.946412
Other religion	1.037	0.964	Yes covered under medical insurance	1.04	0.963796
Mean VIF	1.631		Mean VIF	1.97	

Source: Estimated from the unit-level data of the 75^th^ round of NSS data

#### Results of average marginal effects (AMEs): A sensitivity analysis

Table 5 shows the results of the marginal effects of various socioeconomic and health-related factors influencing the probability of distress financing among households in rural and urban India. In rural areas, female-headed households have a 0.2% greater probability of distress financing than male-headed households do, whereas in urban areas, this probability increases by 0.1%. In terms of Religion, households practicing Islam have a 0.2% greater probability in rural areas but a 1.1% lower probability in urban areas. Households following other religions are 1.8% less likely to engage in distress financing in rural settings but 0.8% more likely in urban areas. Caste status is also significant: Scheduled Tribe (ST) households show a 2.5% lower probability in rural areas and a 0.5% lower probability in urban areas, whereas scheduled caste (SC) households are 2.6% more likely in rural areas and 2.1% more likely in urban areas to depend on distress financing. Other backward class (OBC) households show a 0.8% increase in rural areas and a 3.7% increase in urban areas. Marital status reveals that unmarried households have a 1.2% greater probability in rural areas and 0.3% greater probability in urban areas, whereas widowed or divorced households have a 3.3% greater probability in rural areas and a 1.4% greater probability in urban areas. Households with more than five members are less likely to rely on distress financing, with probabilities decreasing by 2.3% in rural settings and 1.5% in urban settings. Occupational differences show that households engaged in casual or agricultural work have a 2.0% greater probability in rural areas and 3.9% greater probability in urban areas, whereas those engaged in other occupations show a 1.6% greater probability in rural areas and 0.9% greater probability in urban areas. Economic status, measured by consumption expenditure, shows a consistent negative association: the wealthiest households are 3.8% less likely in rural areas and 5.8% less likely in urban areas to use distress financing than the poorest households are. Education level is also relevant. Illiterate households are 1.8% more likely in rural areas and 2.9% more likely in urban areas to depend on distress financing. Households with upper primary education have 1.3% and 2.8% higher probabilities, whereas those with higher secondary education have 0.5% and 2.0% higher probabilities in rural and urban areas, respectively. Healthcare access factors show that using private healthcare facilities increases the probability of distress financing by 0.5% in rural areas and 0.2% in urban areas. Households covered under medical insurance show a substantial increase: 9.2% in rural areas and 5.6% in urban areas. Finally, households with members suffering from chronic ailments face a 6.4% greater probability in rural areas and a 4.3% greater probability in urban areas of relying on distress financing.

**Table 5:** Marginal effects on distress financing in rural and urban India

y=Pr(distress financing) 0.15				y=Pr(distress financing) 0.12		
Variables	Rural			Urban		
	dy/dx	z	P > z	dy/dx	z	P > z
Female	0.002	0	0.002	0.001	0	0.001
Islam	0.002	0	0.002	−0.011	0	−0.011
Others	−0.018	0	−0.018	0.008	0	0.008
ST	−0.025	0	−0.025	−0.005	0	−0.005
SC	0.026	0	0.026	0.021	0	0.021
OBC	0.008	0	0.008	0.037	0	0.037
Unmarried	0.012	0	0.012	0.003	0	0.003
Widowed/Divorced	0.033	0	0.033	0.014	0	0.014
>5 members Household Size	−0.023	0	−0.023	−0.015	0	−0.015
Casual/Agriculture	0.02	0	0.02	0.039	0	0.039
Other occupation	0.016	0	0.016	0.009	0	0.009
Second	−0.005	0	−0.005	−0.004	0	−0.004
Middle	−0.012	0	−0.012	−0.012	0	−0.012
Fourth	−0.035	0	−0.035	−0.029	0	−0.029
Richest	−0.038	0	−0.038	−0.058	0	−0.058
Illiterate	0.018	0	0.018	0.029	0	0.029
Upper Primary	0.013	0	0.013	0.028	0	0.028
Higher Secondary	0.005	0	0.005	0.02	0	0.02
Yes using private facility	0.005	0	0.005	0.002	0	0.002
Yes covered under medical insurance	0.092	0	0.092	0.056	0	0.056
Yes suffering from chronic ailment	0.064	0	0.064	0.043	0	0.043

*Source* Estimated from the unit-level data of the 75th round of NSS data

### Incidence, intensity and inequality of distress financing

Table 6 presents the concentration index (CI) for distress financing related to inpatient care in both rural and urban areas of India for the year 2017. For rural India, the concentration index for distress financing is –0.0030, with a 95% confidence interval from –0.0087 to 0.0027. Since the index value is very close to zero and the confidence interval includes zero, this suggests there is no significant inequality in distress financing by economic status among rural households. In simple terms, both poorer and richer rural households appear equally likely to experience distress financing. For urban India, the concentration index is –0.0723 with a 95% confidence interval from –0.0801 to –0.0644. This finding is statistically significant and negative, indicating that distress financing is more concentrated among economically poorer urban households. In other words, poorer urban families are more likely to rely on borrowing or selling assets to cover healthcare expenses than wealthier urban families are.

**Table 6:** State-level concentration index for distress financing in the case of inpatient care for rural and urban areas in India, 2017

State	Rural		Urban	
	Index Value	Confidence Interval	Index Value	Confidence Interval
JAMMU & KASHMIR	–0.0577	(–0.1202 to 0.0048)	–0.5017	(–0.6027 to –0.4007)
HIMACHAL PRADESH	0.0635	(–0.0045 to 0.1315)	–0.2474	(–0.3914 to –0.1035)
PUNJAB	–0.1851	(–0.2199 to 0.1502)	–0.1300	(–0.1791 to –0.0809)
CHANDIGARH	–0.2793	(–0.3671 to 0.1914)	–0.1232	(–0.2582 to 0.0118)
UTTARANCHAL	–0.2270	(–0.2924 to 0.1617)	0.0051	(–0.0905 to 0.1006)
HARYANA	–0.0204	(–0.0582 to 0.0174)	–0.1965	(–0.2423 to –0.1507)
DELHI	–0.1671	(–0.2453 to 0.0889)	–0.2075	(–0.2622 to –0.1528)
RAJASTHAN	0.01	(–0.0137 to 0.0338)	–0.0859	(–0.1255 to –0.0463)
UTTAR PRADESH	0.0232	(0.0063 to 0.0401)	–0.1430	(–0.1652 to –0.1208)
BIHAR	–0.0225	(–0.0450 to 0.0000)	–0.0849	(–0.1336 to –0.0362)
SIKKIM	–0.2569	(–0.3898 to 0.1240)	0.1219	(–0.5288 to 0.7726)
ARUNACHAL PRADESH	–0.0348	(–0.1201 to 0.0505)	0.1636	(–0.0148 to 0.3419)
NAGALAND	–0.2741	(–0.3422 to 0.2061)	–0.3600	(–0.4821 to –0.2378)
MANIPUR	0.4508	(0.3310 to 0.5706)	0.193	(0.0372 to 0.3488)
MIZORAM	–0.1753	(–0.2953 to 0.0554)	–0.2471	(–0.4396 to –0.0546)
TRIPURA	0.0509	(–0.0119 to 0.1137)	–0.2095	(–0.3243 to –0.0948)
MEGHALAYA	–0.0291	(–0.0885 to 0.0303)	–0.1361	(–0.2606 to –0.0115)
ASSAM	–0.1570	(–0.2080 to 0.1060)	0.0837	(–0.0145 to 0.1829)
WEST BENGAL	–0.0136	(–0.0364 to 0.0092)	0.0123	(–0.0167 to 0.0413)
JHARKHAND	–0.0261	(–0.0547 to 0.0025)	0.0475	(–0.0001 to 0.0951)
ODISHA	0.0631	(0.0341 to 0.0922)	–0.2273	(–0.2932 to –0.1614)
CHHATTISGARH	–0.0434	(–0.0814 to 0.0054)	–0.1607	(–0.2082 to –0.1132
MADHYA PRADESH	–0.0629	(–0.0867 to 0.0390)	–0.0038	(–0.0322 to 0.0245)
GUJARAT	0.05	(0.0090 to 0.0909)	–0.0571	(–0.1013 to –0.0128)
MAHARASHTRA	–0.0319	(–0.0551 to 0.0086)	–0.0722	(–0.1007 to –0.0436)
ANDHRA PRADESH	–0.0187	(–0.0336 to 0.0037)	–0.0796	(–0.1002 to –0.0590)
KARNATAKA	0.0618	(0.0402 to 0.0834)	–0.0862	(–0.1232, –0.0492)
LAKSHADWEEP	0.96	(0.172 to 1.748)	–0.3898	(–0.889 to 0.110)
KERALA	–0.0287	(–0.053 to –0.004)	–0.0413	(–0.072 to –0.010)
TAMIL NADU	0.0183	(–0.002 to 0.039)	–0.0552	(–0.079 to –0.031)
PUDUCHERRY	–0.1968	(–0.28 to –0.11)	–0.0356	(–0.10 to 0.03)
A & N ISLANDS	0.1482	(–0.13 to 0.43)	0.1118	(–0.05 to 0.27)
TELENGANA	0.0296	(0.00 to 0.05)	–0.0812	(–0.11 to –0.05)
India	−0.003	(−0.0086 to 0.0027)	−0.0725	(−0.0801 to − 0.0649)

*Source* Estimated from the unit-level data of the 75^th^ round of NSS data

**Fig. 8:**
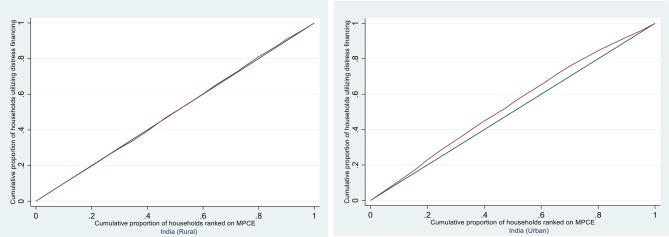
Concentration curves for distress financing (inpatient care) for rural and urban areas in India, 2017. Source Estimated from the unit-level data of the 75th round of NSS data

Figure 8 shows the concentration curves (CC) for distress financing in inpatient care across rural and urban areas in India. The position of the CC relative to the line of equality provides a visual representation of inequality in distress financing. In urban areas, if the CC lies significantly below the line of equality, it indicates that lower-income households are more affected by distress financing than wealthier households are. Conversely, if the CC for rural areas is closer to or above the line of equality, it suggests that wealthier households may not be as heavily impacted by distress financing.

## Discussions

The findings of this study align with and extend earlier national-level evidence. For instance [14, 21] also reported that low-income, socially disadvantaged, and uninsured households are more prone to distress financing. However, by using the NSS 75th round, our analysis captures post–Ayushman Bharat patterns, revealing that even insured households—especially in rural areas—continue to face financial distress. This contrasts with the expected protective effect of insurance and suggests implementation gaps in coverage, awareness, and reimbursement processes. Similar findings were observed in recent state-level studies [10, 11], emphasizing the need for stronger financial risk protection and more equitable public healthcare investment across states. For example, sub-Saharan Africa reports persistently high OOP levels (with recent figures near 40.8%), whereas countries such as Argentina and Tanzania also exhibit widespread use of distress financing owing to similarly high OOP and underdeveloped financial protection systems.

As in India, the main drivers of distress financing in other developing settings such as Argentina, Tanzania, and Bangladesh, are low public health investment, limited or fragmented insurance coverage, and heavy dependence on often-unregulated private health services. In China, substantial reforms and the expansion of public health insurance have successfully reduced OOP expenditures from over 64% in 2001 to 28.6% in 2021, directly decreasing the need for distress financing. In contrast, India’s slower progress in risk pooling and public spending means that large sections of the population, especially rural and marginalized groups, remain exposed to financial shocks from illness.

Nanda & Sharma [11] highlighted that India’s public health expenditure remains among the lowest globally, resulting in limited access to quality public healthcare services. Consequently, a large proportion of the population is compelled to seek care from private providers, where costs are substantially higher and often unregulated. This reliance on private healthcare significantly increases the risk of catastrophic health spending and distress financing, especially among poorer households and socially disadvantaged groups. Furthermore [21], emphasized that government-sponsored health insurance schemes, while expanding coverage, often fail to provide adequate financial protection, particularly for outpatient care and chronic illnesses, which constitute a major share of household health expenses. The combination of these factors—low public investment, insufficient insurance, and high private sector costs—contribute to the ongoing vulnerability of Indian households to health-related financial shocks, as reflected in the country’s OOP share remaining much higher than the global average [11, 21].

The analysis reveals that the incidence of distress financing is far from uniform across the country. In the present study, rural households in states such as Uttar Pradesh, West Bengal, Maharashtra, and Rajasthan exhibit the highest reliance on distress financing, with rates as high as 19.1% in rural Uttar Pradesh. According to a study by [35], nearly 69–70% of the health infrastructure in these states is under private ownership. Owing to the limited availability of public healthcare services, people are often compelled to turn to private facilities, which leads to financial strain. The cost of treatment in private facilities is significantly greater than that in government facilities. However, owing to perceived or real inadequacies in public healthcare, many households still seek private care, increasing their financial burden [36]. In contrast, states such as Sikkim, Nagaland, and Goa report minimal distress financing, suggesting that stronger public health infrastructure and more effective financial protection mechanisms can mitigate the risk of catastrophic health expenditures. These findings are consistent with earlier studies, which highlighted the role of regional health system performance and socioeconomic development in shaping household vulnerability to health shocks [37, Sangar 2019]. Poorer states and regions, especially those with large populations of socially and economically disadvantaged groups, are more vulnerable to health shocks. These households often lack savings or access to affordable formal credit, making them more likely to borrow at high interest or sell assets to pay for healthcare [38].

Despite better healthcare infrastructure in urban settings, financial protection remains insufficient, particularly for low-income groups. This study reveals that urban areas in states such as Maharashtra, Tamil Nadu, and Delhi have high rates of distress financing, likely due to greater reliance on private healthcare services and high out-of-pocket costs. Such cost inflation directly impacts urban households, as many households are forced to borrow money or sell assets to pay for medical care, especially in the absence of adequate health insurance coverage [39]. This urban paradox points to the limitations of insurance schemes and public sector reach, even in more developed settings.

The negative values of the concentration index (CI) and the positioning of the concentration curve above the line of equality both confirm that the financial burden is regressive, falling most heavily on the poorest households. This finding echoes the broader literature on health financing in low- and middle-income countries, where high OOP expenditures and insufficient insurance coverage remain primary drivers of financial vulnerability among low-income and marginalized groups [Kane et al. 2023; 10].

The multivariate logistic regression analysis in the study revealed that, households in the lowest wealth quintiles, those with lower educational attainment, and those belonging to the SC and ST groups are significantly more likely to resort to distress financing. These groups are more exposed to distress financing because they generally have lower incomes, fewer savings, and limited asset ownership, making it harder to absorb unexpected health costs [Kumar et al. 33, 25]. Compared with male-headed households, female-headed households are less likely to resort to distress financing, households possibly due to more cautious financial management practices, as supported by [21]. Education emerges as a key protective factor, with illiterate households being more likely to depend on distress financing, underscoring the role of financial literacy and awareness in shaping coping strategies [40].

The analysis also reveals a nuanced picture of health insurance. While insured urban households experience reduced dependency on distress financing, rural insured households remain vulnerable, suggesting that insurance schemes are less effective in rural areas. High out-of-pocket expenses persist in rural settings, leading to financial distress despite insurance coverage [41]. The findings suggest that despite being enrolled in health insurance programs such as the Rashtriya Swasthya Bima Yojana (RSBY) and similar schemes, some individuals do not utilize their health cards due to a lack of awareness or simply forgetting to use them. Certain studies have highlighted that although insurance companies have conducted widespread awareness campaigns, the emphasis has been largely on explaining what the scheme entails and who qualifies for it, with minimal focus on how to use the card and access the benefits [42]. Conversely, other research indicates that even when people use health insurance, the coverage provided is often inadequate to offset the high out-of-pocket expenses associated with noncommunicable diseases [43]. Many insurance schemes, such as Ayushman Bharat and state-level programs, cover inpatient care and exclude outpatient services, diagnostics, and medicines, which constitute a significant portion of rural healthcare expenses [44].

## Conclusion

The present study confirms that distress financing for inpatient healthcare expenses remains a significant and persistent issue in India, particularly among rural households, lower-income groups, and socially disadvantaged communities such as Scheduled Castes (SC), Scheduled Tribes (ST), and Other Backward Classes (OBC). Despite a visible reduction in out-of-pocket (OOP) health expenditures over the past two decades, these improvements have not translated into equitable financial protection across all socioeconomic groups and regions. States such as Uttar Pradesh, Maharashtra, and West Bengal continue to show the highest levels of distress financing, which underlines existing disparities in healthcare access, public healthcare infrastructure, and insurance coverage.

In summary, distress financing for inpatient healthcare remains a serious concern despite declining out-of-pocket expenses in India. The persistence of high reliance on borrowings and asset sales among poor, rural, and socially disadvantaged households underscores unequal benefits of financial protection schemes. To address this, policymakers should focus on:

(i) strengthening Ayushman Bharat and state-level health insurance to ensure timely and adequate coverage; (ii) expanding the scope of insurance to include outpatient and chronic illness care; (iii) improving awareness and utilization of existing schemes, particularly in rural regions; and (iv) increasing public health expenditure to reduce dependence on costly private care. These measures are essential to achieving Universal Health Coverage (UHC) and preventing health-related impoverishment among India’s most vulnerable populations. In these contexts, distress financing also arises from a combination of high OOP healthcare expenses, insufficient public health investment, fragmented insurance systems, and heavy reliance on unregulated private healthcare providers. Moreover, examples such as China demonstrate that comprehensive public health reforms—focusing on expanding insurance coverage, improving public healthcare services, and regulating healthcare costs—can reduce household reliance on distress financing.

On the basis of study findings, several specific suggestions emerge. First, there is an urgent need to expand the coverage and depth of public health insurance schemes in India, ensuring that cover not only inpatient care but also outpatient services, medicines, and diagnostic costs. Second, public healthcare infrastructure, especially in rural and high-burden states, must be strengthened to reduce reliance on costly private services. Third, targeted financial protection mechanisms should be introduced for households in the lowest economic quintiles and for vulnerable social groups such as the SC and ST communities. Fourth, improving health insurance literacy is essential so that enrolled households can effectively benefit from health insurance. Finally, there should be stronger regulation of private healthcare service pricing to control out-of-pocket expenses and protect households from financial distress.

In general terms, distress financing reflects the failure of health systems to provide equitable financial protection, particularly where public healthcare infrastructure is weak and private healthcare dominates. These dynamics apply across many countries, especially in Asia, Africa, and Latin America, where similar socioeconomic vulnerabilities intersect with health financing deficiencies. This study has several limitations. It is based on cross-sectional data, so it cannot show cause and effect relationships or long-term trends. There may be underreporting of distress financing, especially through informal borrowing or selling assets, as people may not fully disclose such information. The study records whether households have health insurance but does not check whether they actually use it or if it provides enough financial support. Additionally, the focus is only on inpatient care expenses; outpatient care and long-term health costs are not included. Other factors, such as healthcare quality or local service availability are not covered in this analysis.

## Data Availability

The datasets analyzed for this study are publicly available from the National Sample Survey Office (NSSO) 75th Round, 2017–18 and WHO Global Health Expenditure Database. Data can be accessed from https://microdata.gov.in/nada/index.php/catalog/152 and WHO data can be accessed from https://apps.who.int/nha/database/Select/Indicators

## Abbreviations

OOP: Out-of-pocket expenditure
DF: Distress financing
NSSO: National Sample Survey Organization
OOPE: Out-of-pocket expenditure
NSS: National Sample Survey
SC: Scheduled Caste
ST: Scheduled Tribe
OBC: Other Backward Classes
MPCE: Monthly per capita consumption expenditure
CI: Concentration index
CC: Concentration curve
FSU: First-stage unit
LR: Logistic regression
VIF: Variance inflating factor
RSBY: Rashtriya Swasthya Bima Yojana

## Footnotes

1 Distress financing, defined as resorting to borrowings, sale of assets, or help from friends or relatives to cover hospitalization costs, reflects the inadequacy of financial protection in the health system.
